# Defect Analysis in a Long-Wave Infrared HgCdTe Auger-Suppressed Photodiode

**DOI:** 10.3390/s24113566

**Published:** 2024-06-01

**Authors:** Małgorzata Kopytko, Kinga Majkowycz, Krzysztof Murawski, Jan Sobieski, Waldemar Gawron, Piotr Martyniuk

**Affiliations:** 1 Institute of Applied Physics, Military University of Technology, 2 Kaliskiego St., 00-908 Warsaw, Poland; malgorzata.kopytko@wat.edu.pl (M.K.); kinga.majkowycz@wat.edu.pl (K.M.); krzysztof.murawski01@wat.edu.pl (K.M.); jsobieski@vigo.com.pl (J.S.); piotr.martyniuk@wat.edu.pl (P.M.); 2 Vigo Photonics S.A., 129/133 Poznańska St., 05-850 Ożarów Mazowiecki, Poland

**Keywords:** infrared detectors, HgCdTe, Auger suppression, defect levels, DLTS, photoluminescence

## Abstract

Deep defects in the long-wave infrared (LWIR) HgCdTe heterostructure photodiode were measured via deep-level transient spectroscopy (DLTS) and photoluminescence (PL). The n^+^-P^+^-π-N^+^ photodiode structure was grown by following the metal–organic chemical vapor deposition (MOCVD) technique on a GaAs substrate. DLTS has revealed two defects: one electron trap with an activation energy value of 252 meV below the conduction band edge, located in the low n-type-doped transient layer at the π-N^+^ interface, and a second hole trap with an activation energy value of 89 meV above the valence band edge, located in the π absorber. The latter was interpreted as an isolated point defect, most probably associated with mercury vacancies (V_Hg_). Numerical calculations applied to the experimental data showed that this V_Hg_ hole trap is the main cause of increased dark currents in the LWIR photodiode. The determined specific parameters of this trap were the capture cross-section for the holes of *σ_p_* = 10^−16^–4 × 10^−15^ cm^2^ and the trap concentration of *N_T_* = 3–4 × 10^14^ cm^−3^. PL measurements confirmed that the trap lies approximately 83–89 meV above the valence band edge and its location.

## 1. Introduction

Long-wave infrared (LWIR) imaging covers wavelengths ranging from 8 μm to 14 μm. LWIR photodetectors find many practical applications, since objects at a close-to-room temperature emit thermal radiation exactly in this range. For example, the radiation emitted by the human body reaches peak values at wavelengths between 9 and 10 μm. Beyond thermal imaging, since LWIR radiation has better transmission properties through atmospheric conditions, such as fog, smoke, and dust, this range is also used in free-space telecommunications. Another common application of LWIR detectors is spectroscopy to detect toxic and hazardous substances.

However, to reach high sensitivity, LWIR photodetectors must be cooled to cryogenic temperatures. This is due to the large number of intrinsically generated carriers that have a low-energy threshold due to the narrow semiconductor bandgap (of about 0.1–0.15 eV). Many technological approaches are undertaken to minimize the thermal generation in the detector active element and increase its operating temperature without reducing the quantum efficiency, including the following: (i) a reduction in the detector volume with the use of optical concentrators, back-side reflectors, or optical resonant cavities; (ii) the suppression of Auger thermal generation with non-equilibrium effects; and (iii) material doping and structural defect optimization. Most often, all of the above solutions are used simultaneously.

Mercury cadmium telluride (HgCdTe) is a narrow-gap semiconductor that enables the development of so-called high-operating-temperature (HOT) photodetectors [1,2,3,4,5]. This is ensured by its specific advantages, such as being direct and tunable in a wide energy range bandgap, the possibility of obtaining both low and high carrier concentrations, with the possibility of the sophisticated multilayer heterostructures growing.

The use of the n^+^-P^+^-π-N^+^ photodiode structure (the capital letter denotes a wider-gap semiconductor, the upper index “+” denotes heavy doping, and π denotes low p-type doping) makes it possible to suppress the Auger generation under non-equilibrium operating conditions [6,7,8]. Under a reverse bias, intrinsically generated electrons are excluded from the absorber region near the P^+^-π junction because they cannot be injected from the P^+^ layer and are also extracted from the absorber region near the π-N^+^ junctions due to a positive electrode being connected to the N^+^ layer. As a consequence, the hole concentration in the π absorber decreases to the acceptor doping level.

Moreover, the use of optical immersion increases the optical size of the detector compared to its actual physical size. Since the metal–organic chemical vapor deposition (MOCVD) technique allows the epitaxial layers to be deposited on lattice-mismatched substrates, the immersion lens is formed directly from the GaAs substrate using numerically controlled micromachining [9]. However, the lattice mismatch between the GaAs substrate and the CdTe buffer is a source of dislocation that can extend deep into the HgCdTe layer. Dislocations, together with point defects located in the absorber region and heterojunctions, are a possible cause of the increased Shockley–Read–Hall (SRH) generation.

Several literature studies have been published on the issues surrounding Auger-suppressed HgCdTe photodiodes [10,11,12,13,14,15,16,17,18]. Among other things, the numerical modeling of predicted parameters [10,11], engineering steps (aimed at optimizing the photodiode architecture [12] and noise [13]), and time response studies (which have shown that the highest detectivity is obtained close to the negative resistance conditions at the expense of a long time constant [14,15,16]) were performed. It was also shown that, with good-quality material and with low concentrations of residual impurities and defects, the HgCdTe photodiode can operate in conditions associated with the complete depletion of the absorber [19,20]. As a result, using the exclusion and extraction phenomena, an effect similar to that reached when cooling the detector is achieved, i.e., by reducing the carrier concentration in the absorber, Auger generation and recombination are reduced. As a result, detectivity increases. However, they do not present measurements of deep defects in heterostructure LWIR HgCdTe photodiodes or demonstrations of their impact on the operation of Auger-suppressed photodiodes.

This paper seeks to identify and characterize the SRH centers that occur in a LWIR HgCdTe heterostructure photodiode. The specific parameters characterizing the SRH defect (such as the activation energy of the trap level, *E_T_*; the hole capture cross-section, *σ_p_*; and the trap-level concentration, *N_T_*) were determined using deep-level transient spectroscopy (DLTS). The trap-level energy was also confirmed by the photoluminescence (PL) measurements. Finally, theoretical analysis was performed using the APSYS simulation package.

## 2. Materials and Methods

The HgCdTe epitaxial layer was grown by following the metal–organic chemical vapor deposition (MOCVD) method on a 2” GaAs substrate. The AIX-200 Aixtron MOCVD system was used for growth purposes, and a horizontal reactor with laminar gas flow and process pressures ranging from 50 to 1000 mbar was implemented. Growth was carried out at a pressure rate of 500 mbar, and hydrogen was used as a carrier gas. Diisopropyl telluride (DIPTe) and dimethyl cadmium (DMCd) were used as precursors, while tris-dimethylamino-arsenic (TDMAAs) and ethyl iodide (EI) were used as acceptor and donor dopant sources, respectively. Growth was carried out on 2-inch, epi-ready, semi-insulating (100) GaAs substrates, oriented 2° off toward the nearest <110>. First, the CdTe buffer layer was deposited directly on GaAs, which reduces the stress caused by a 14% crystal lattice misfit between HgCdTe and the GaAs substrate. The HgCdTe epilayers were deposited using the inter-diffusion multilayer process (IMP) method [21,22], and increased composition and thickness uniformities were obtained using the Aixtron gas foil rotation technique. The HgCdTe was grown at 350 °C and a mercury source was kept at a temperature of 160–220 °C. In situ annealing was carried out under mercury-rich conditions to reduce the residual mercury vacancy concentration and to increase the uniformity of the HgCdTe heterostructures, which allowed ex situ annealing to be avoided. The HgCdTe growth with the MOCVD technique was more extensively presented in Refs [21,22,23,24].

**Figure 1:**
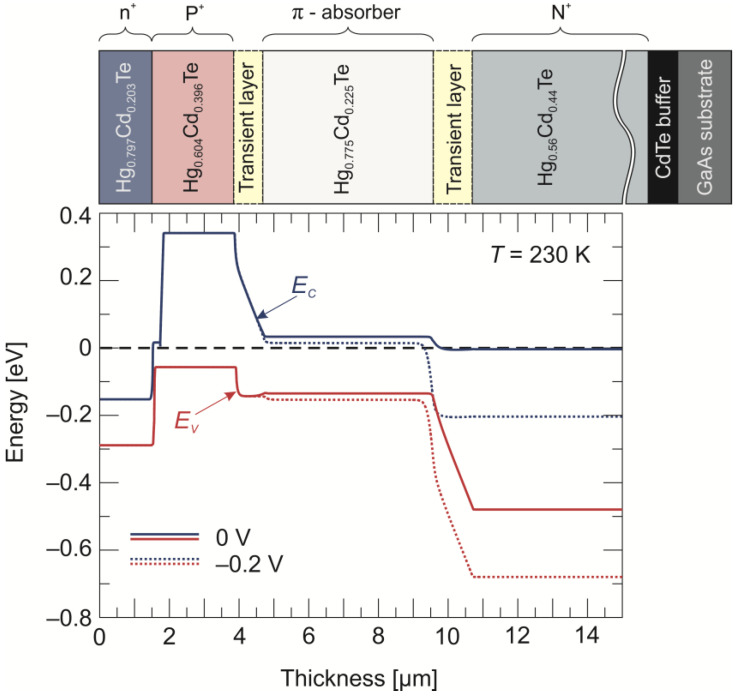
Diagram showing the calculated band along the cross-section of the LWIR n^+^-P^+^-π-N^+^ HgCdTe photodiode under 0 and −0.2 V bias voltages and 230 K. *E_C_* denotes the conduction band edge and *E_V_* denotes the valence-band edge.

The device that we used for analysis is a four-layer n^+^-P^+^-π-N^+^ photodiode with a ~5 µm thick p-type (*N_A_* = 3 × 10^15^ cm^−3^) narrow-gap absorber (*x* = 0.225), sandwiched between wide-gap P^+^ (*x* = 0.396, *N_A_* = 5 × 10^17^ cm^−3^) and N^+^ (*x* = 0.44, *N_D_* = 2 × 10^17^ cm^−3^) layers. The top n^+^ layer was used to provide low-resistance ohmic contact. The CdTe buffer layer was used in order to compensate the lattice mismatch between the HgCdTe epilayer and the GaAs substrate. The photodiode was nominally designed to operate at 230 K. The calculated band structure drawn along the cross-section of the analyzed n^+^-P^+^-π-N^+^ HgCdTe photodiode is presented in Figure 1. The transient-layer parameters were conditioned using interdiffusion processes during the MOCVD growth period at 350 °C. The space charge region was mainly deposited in the transient layer and extended into the π absorber.

Prior to the device fabrication step, the upper layers were etched off to expose the absorber in order to obtain the PL measurements. The PL signal was collected using the FTIR Bruker Vertex 70v spectrometer in the step-scan mode. A 640 nm line, mechanically chopped with a 10 kHz frequency laser, was used to generate excess carriers. The PL signal was detected with a liquid-nitrogen-cooled MCT D315 photodiode with a 25 μm cut-off and analyzed with a lock-in amplifier. The measurements were made in a vacuum to avoid any impacts of IR absorption lines in the air.

Then, the electrical and optical measurements of the device were taken. For this purpose, the 400 μm × 400 μm mesa-type structures were defined using photolithography and wet-etched in a Br:HBr (1:100) diluted in a deionized water (50:50:1 Br:HBr:H_2_O) solution. Au metallic contacts were deposited on the top n^+^ and the bottom N^+^ layers. Quantum efficiency (QE) measurements were performed in a back-side-illuminated photodiode using a FTIR Perkin Elmer spectrometer (Spectrum 2000). A complete hardware and software Semetrol system (Semetrol, LCC, Midlotjian, VA, USA) was used for the DLTS study. A double “box-car” was used to define the rate window (RW) with an assumed value of 17 s^−1^. A reverse-bias voltage of *V_R_* = –1 V was used to establish a steady-state depletion width. Next, a positive fill pulse of *V_p_* = 0.3 V was used to decrease the depletion region width. The measurement was taken at a frequency of 200 kHz and a variable filling pulse width (*t_p_* = 0.1–10 ms). The detector was mounted in a helium closed-cycle cryostat which ensured temperature stabilization within the range of 50 K to 300 K. Dark current density–voltage (*J*–*V*) plots were also collected using the Semetrol system in of bias voltage range of 0.2 V to −0.8 V.

Theoretical modelling was performed using the commercially available APSYS platform (Crosslight Inc., Burnaby, Canada). The performance of the detectors was assessed, taking into account a wide spectrum of GR mechanisms (Auger, SRH, and tunneling processes).

## 3. Results

**Figure 2:**
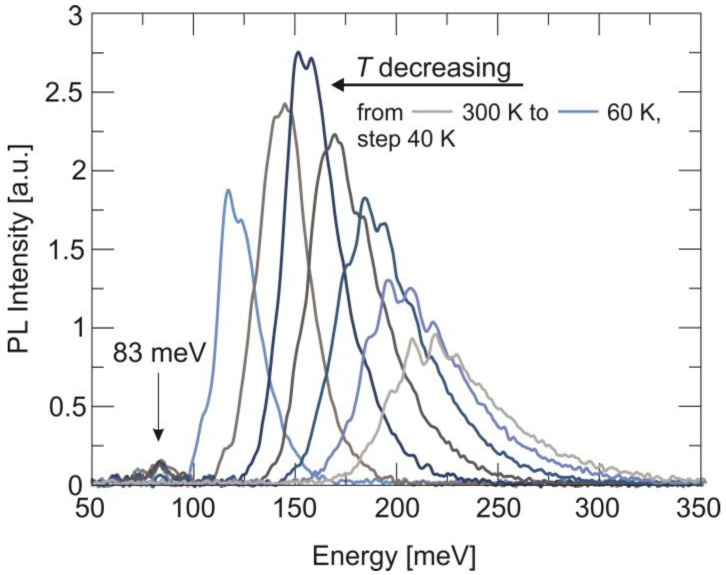
PL spectra of the HgCdTe (*x_Cd_* = 0.225) π absorber collected for selected temperatures between 60 K and 300 K. The PL peak around 83 meV is connected with the defect-related transition.

PL is a powerful type of optical spectroscopy that is widely used to study the optical properties of semiconductors, including defect states. Figure 2 shows that the PL spectra of HgCdTe (*x_Cd_* = 0.225) π absorbers were collected for selected temperatures between 60 K and 300 K, and under low-excitation-intensity regimes (4.5 W/cm^2^). The PL spectrum is dominated by the peak that originates from the main band-to-band transition. This peak with decreasing temperature is blue-shifted according to the theoretical Hansen relationship [25] for the HgCdTe energy gap. The second peak at the lower-energy side has a constant position with temperature, which is characteristic of defect states. All PL transitions that take place through another level (defect or dopant) inside the bandgap cannot take when this level lies below the Fermi level. Due to the large difference in the mobility of electrons and holes in HgCdTe, the Fermi level at high temperatures lies close to the edge of the conduction band, as well as in p-type material (see also Figure 1). When the temperature decreases, the Fermi level also decreases, and when it is below the defect level, this level can be occupied by an electron [26]. The PL transition for this defect peak visible in temperatures at *T* < 180 K suggests that, first, the electron non-radiatively passes from the conduction band to the defect level, and it then falls to the valence band, emitting a photon with an energy value of about 83 meV. Therefore, the determined energy position of the trap level relative to the valence band is 83 meV.

**Figure 3:**
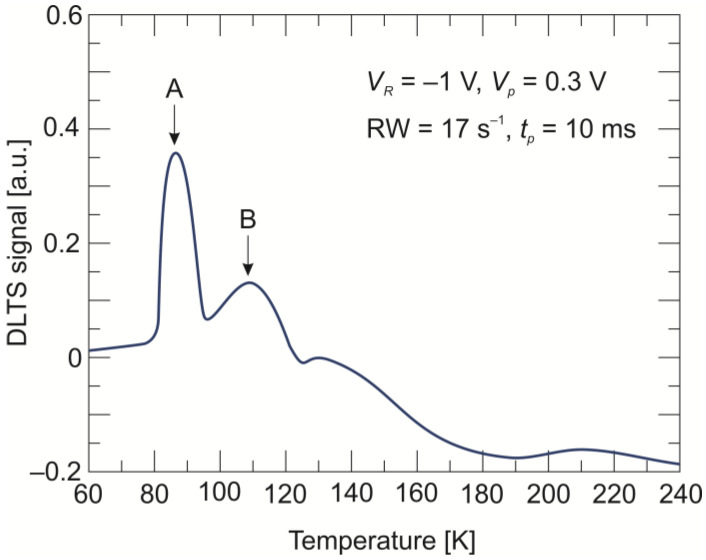
DLTS signal recorded in the temperature range between 60 K and 240 K for the LWIR n^+^-P^+^-π-N^+^ HgCdTe photodiode.

The second method that can be used to investigate defect levels in semiconductors is DLTS. In our previous papers [27,28], we developed and described a method for measuring and analyzing DLTS data in multilayer heterostructure photodiodes. The DLTS signal, recorded when a positive fill pulse of *V_p_* = 0.3 V was applied and reverse-biased with a *V_R_* = −1 V photodiode junction, is shown in Figure 3. The measurement was made at a frequency of 200 kHz and a filling pulse width of *t_p_* = 10 ms. Because of the specific design of the π-N^+^ heterostructure junction (with a low p-type-doped absorber, a low n-type-doped transient layer, and a heavy n-type-doped N^+^ layer), the space charge region is mainly deposited in the transient layer and extends into the π absorber. Two positive peaks (marked as A and B) that correspond to the majority-carrier traps were observed in the temperature range between 70 K and 120 K.

**Figure 4:**
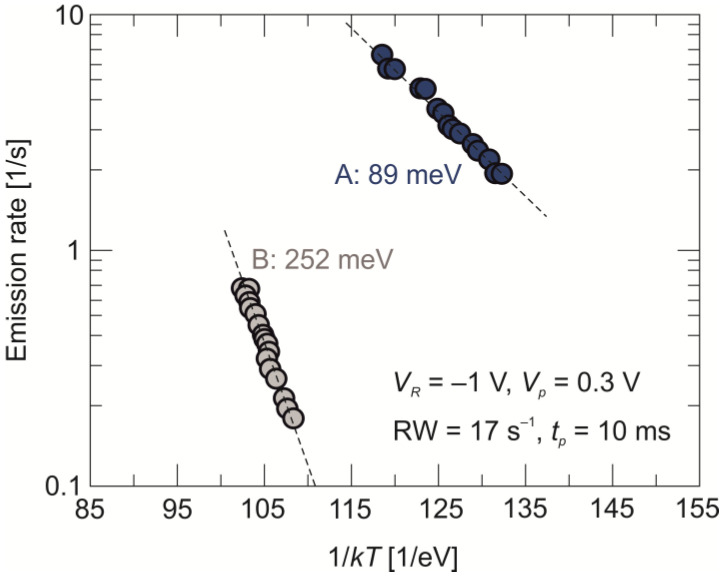
Arrhenius plot of the emission rate corresponding to the appropriate DLTS peaks.

An emission rate versus 1/*kT* (the Arrhenius plot), where *k* is the Boltzmann constant and *T* is the temperature, corresponding to the appropriate DLTS peaks A and B, is presented in Figure 4. An Arrhenius plot of the emission rate enables a straight-forward determination of two parameters which characterize deep-trap levels: the activation energy that is determined from the slope of the curve and the capture cross-section for carriers from the intersection with the ordinate axis. Semetrol software (Semetrol, LCC, Midlotjian, United States) provides these parameters obtained by means of the standard least-squares fitting procedure.

As mentioned earlier, both peaks correspond to the majority-carrier traps. If peak A is related to a majority-carrier trap within the π absorber bandgap, it can be considered a hole trap with an activation energy value of *E_V_* + 89 meV. This value is very close to the one determined earlier from the PL measurements. The energy level for thermal emission estimated for the peak B is 252 meV and exceeds the energy gap value of the absorber (*E_g_* = 121 meV at 77 K and *E_g_* = 189 meV at 300 K). Assuming that this trap is located in the low n-type-doped transient layer at the π-N^+^ interface, it can therefore be considered as a majority electron trap with an activation energy value of *E_C_* − 252 meV.

From the point of view of the device’s operation, a deep defect in the absorber region, i.e., originating from peak A, may have a greater impact on the dark currents. By analyzing the shape of the peak, it is possible to determine the type of defect based on the notion that narrow and symmetrical peaks are characteristic of isolated point defects. Moreover, the type of defect can be examined by analyzing carrier capture kinetics. 

**Figure 5:**
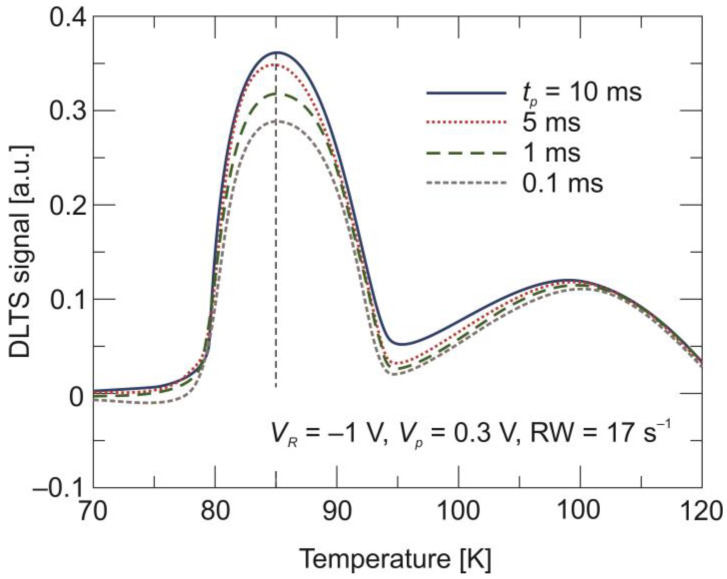
DLTS signal for the LWIR n^+^-P^+^-π-N^+^ HgCdTe photodiode recorded for selected values of the filling pulse width.

**Figure 6:**
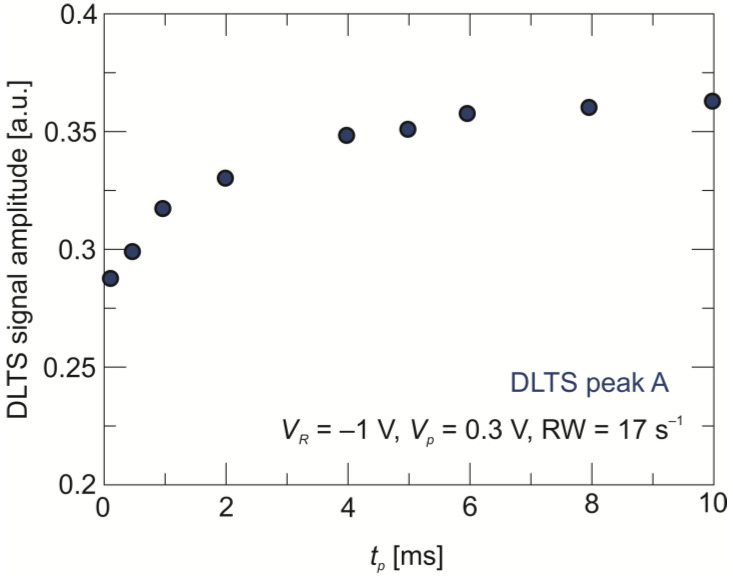
Dependence of DLTS signal amplitude on the filling pulse width for trap A.

Figure 5 shows the DLTS signal recorded for different values of the filling pulse width, *t_p_*, ranging from 0.1 ms to 10 ms. Firstly, peak A does not change the position with the change in filling pulse duration. Secondly, as presented in Figure 6, the amplitude of the peak A DLTS signal reaches the saturation at *t_p_* = 6 ms. This type of behavior allows peak A to be attributed to a point defect. It should be mentioned here that a defect at the 80–100 meV level was identified in HgCdTe deposited with various methods and assigned to mercury vacancies (V_Hg_) [29,30,31,32,33]. 

We also determined the capture cross-section of this V_Hg_ level for holes to be *σ_p_* = 4 × 10^−15^ cm^2^. The apparent concentration of the V_Hg_ level trap amounts to *N_T_* = 3 × 10^14^ cm^−3^.

**Figure 7:**
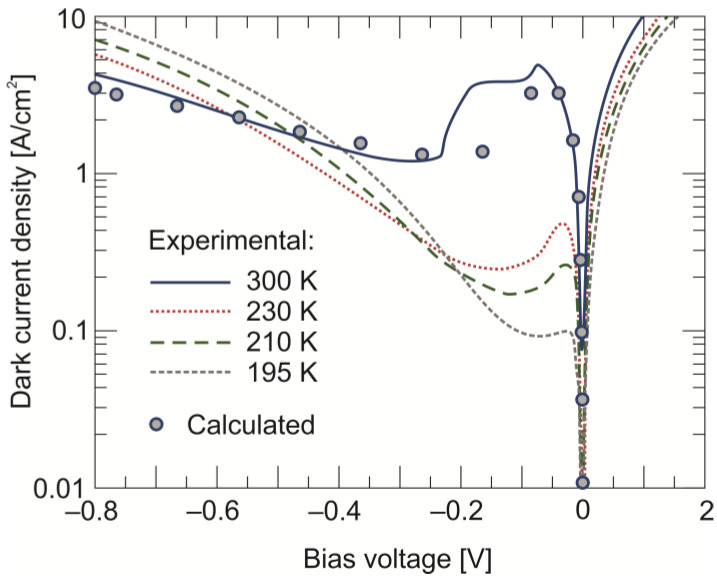
Dark current density vs. voltage characteristics measured at four temperatures for the LWIR n^+^-P^+^-π-N^+^ HgCdTe photodiode. Pointed plot shows the theoretical fit to the experimental results at 300 K.

Figure 7 shows the set of dark *J*–*V* plots measured at four temperatures between 190 K and 300 K. The photodiode is characterized by Auger suppression, most visible at room temperature but starting at higher reverse bias voltages. In an ideal detector, the current after suppressing the Auger generation should be saturated. The visible increase in dark currents with a further increase in reverse voltage is caused by the undesirable SRH mechanism. By assuming the trap parameters determined in previous measurements, the experimental curve at 300 K was fitted using numerically calculated data. A good fit was achieved, assuming that one defect lies within the absorber bandgap with the parameters collected in Table 1. Similar experimental and theoretical values show that the above method is effective for the correct identification and characterization of defects in heterostructure photodiodes.

**Figure 8:**
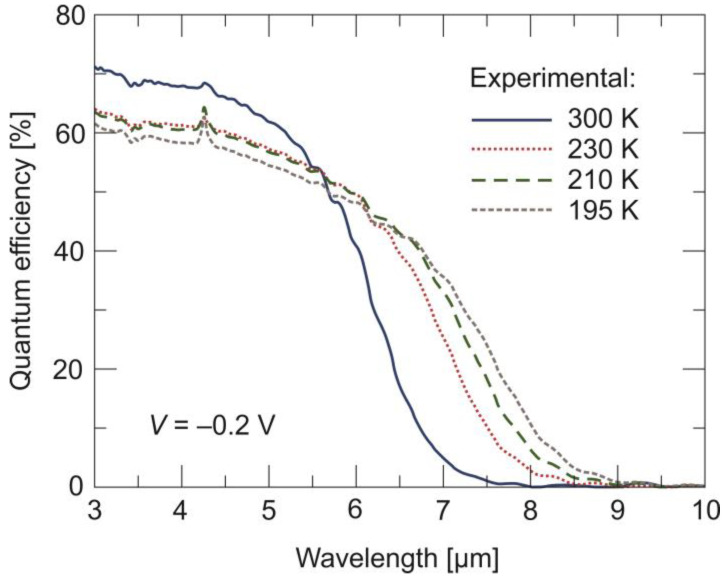
Quantum efficiency vs. wavelength measured at four temperatures and a –0.2 V bias voltage for the back-side-illuminated LWIR n^+^-P^+^-π-N^+^ HgCdTe photodiode.

Figure 8 shows the QE spectral response measured at four temperatures between 190 K and 300 K, as well as a –0.2 V bias voltage for the back-side-illuminated 400 μm × 400 μm mesa detector. The cut-off wavelength at 190 K is ~8 μm and is blue-shifted to ~6.5 μm at 300 K. The photodiode has a similar maximum QE of 60% at a low temperatures and a slightly higher QE of 65% at 300 K. Moreover, the spectral roll-off is reasonably sharp at 300 K. Smaller peak QE and a more soft spectral roll-off at lower temperatures indicate the use of an overly thin absorber compared to the carriers’ diffusion length.

**Figure 9:**
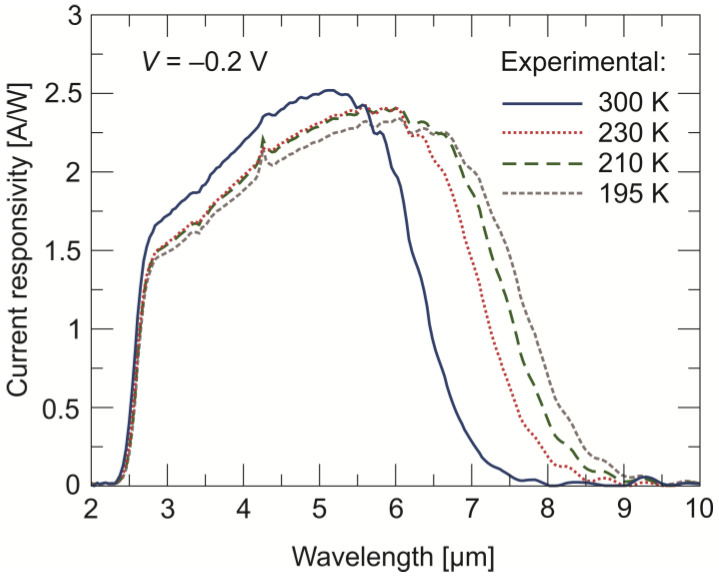
Current responsivity vs. wavelength measured at four temperatures and a –0.2 V bias voltage for the back-side-illuminated LWIR n^+^-P^+^-π-N^+^ HgCdTe photodiode.

A QE of 60–65% translates to high current responsivity (*R_i_*) values. Figure 9 shows the spectral *R_i_* of the photodiode measured at four temperatures. The highest maximum *R_i_* is at room temperature and is equal to ~2.5 A/W. The *R_i_* allows one of the most important parameters of the infrared detector, i.e., normalized detectivity, marked as (D*, in units of cmHz^1/2^/W, also called as Jones), to be estimated.

D* determines the signal-to-noise ratio of the detector, normalized to the square root of the detector area (*A*) and the measurement bandwidth (Δ*f*) [34]:

(1) $$D^{*}=\frac{R_{i}}{i_{n}}\sqrt{A\Delta f}=\frac{R_{i}}{\sqrt{2q\left(J_{d}+2J_{s}\right)}},$$

where *i_n_* is the noise current; *q* is the electric charge; *J_d_* is the density of current passing through the detector, expressed as $J_{d}=J_{s}\left[\exp\left(qU/k_{B}T\right)-1\right]$; *J_s_* is the saturation current density; *k_B_* is the Boltzmann constant; and *T* is the absolute temperature.

If the photodiode operates under reverse bias, where *J_d_* tends to –*J_s_*, D* can be described as the following:

(2) $$D^{*}=\frac{R_{i}}{\sqrt{2qJ_{s}}}.$$

In practice, for the analyzed non-equilibrium photodiode, we can also determine D* from the following relationship:

(3) $$D^{*}=\frac{R_{i}}{\sqrt{2qJ_{d}+\frac{4kT}{R_{d}A}}},$$

where *R_d_* is the dynamic resistance of the photodiode.

For the analyzed non-equilibrium photodiode operating under reverse bias, in the current saturation region where *R_d_* is very large, we can obtain the following:

(4) $$2qJ_{d}\gg \frac{4kT}{R_{d}A},$$

and then we can accept it as the following:

(5) $$D^{*}=\frac{R_{i}}{\sqrt{2qJ_{d}}}.$$

**Figure 10:**
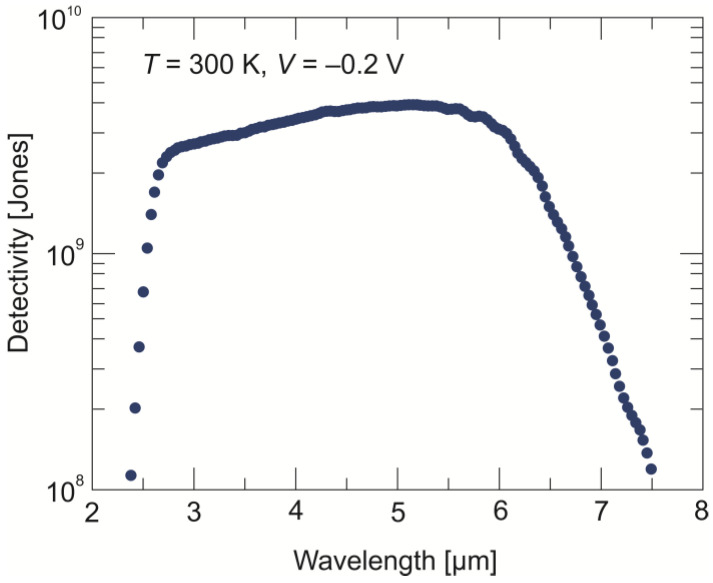
Detectivity vs. wavelength determined at 300 K and a –0.2 V bias voltage for the back-side-illuminated LWIR n^+^-P^+^-π-N^+^ HgCdTe photodiode.

The normalized spectral detectivity of the photodiode operating at a temperature of 300 K and a −0.2 V bias voltage is presented in Figure 10. A reduction in the dark current from ~5 A/cm^2^ to ~1.1 A/cm^2^ causes the non-equilibrium photodiode to reach a maximum D* of 4 × 10^9^ cmHz^1/2^/W.

## 4. Conclusions

In summary, despite Auger suppression, the SRH-dominated dark current was observed in the LWIR n^+^-P^+^-π-N^+^ HgCdTe photodiode grown using MOCVD on the GaAs substrate. DLTS measurements, performed in the temperature range between 60 K and 240 K, revealed two defects: an electron trap with an activation energy value of *E_C_* − 252 meV, located in the low n-type-doped transient layer at the π-N^+^ interface, and a hole trap with an activation energy value of *E_V_* + 89 meV, located in the π absorber. Numerical calculations fitted to experimental data showed that the increased dark current is attributed to the existence of the deep defect lying within the narrow-gap absorber. The measurements of capture kinetics for this trap indicated the stability of the DLTS peak maximum with an increasing filling pulse duration and a distinct saturation for long filling pulse times, both typical for isolated point defects. In the case of the HgCdTe absorber, the hole trap is most probably associated with V_Hg_. The determined specific parameters of this trap were the capture cross-section for holes (*σ_p_* = 10^−16^–4 × 10^−15^ cm^2^) and the trap concentration (*N_T_* = 3–4 × 10^14^ cm^−3^). The trap was found to lie approximately 83–89 meV above the valence band edge and its location was also confirmed by the PL measurement.

## Figures and Tables

**Table 1:** V_Hg_ trap parameters determined from the PL and DLTS measurements and values assumed in numerical calculations.

Parameter	Determined	Assumed
Defect identification	V_Hg_	
Activation energy, *E_T_* – *E_V_* [meV]	83 (PL)	86
	89 (DLTS)	
Trap concentration, *N_T_* [cm^−3^]	3 × 10^14^	3.4 × 10^14^
Hole capture cross-section, *σ_p_* [cm^2^]	4 × 10^−15^	1 × 10^−16^

## Data Availability

Data are contained within the article.
